# Bis[2-((4,6-dimethyl­pyrimidin-2-yl){2-[(4,6-dimethyl­pyrimidin-2-yl)sulfan­yl]eth­yl}amino)­eth­yl] disulfide

**DOI:** 10.1107/S1600536810035245

**Published:** 2010-09-08

**Authors:** Guo-Qing Wang, Cong-Hui Ma, Wen-Ge Li, Xiao-Feng Li, Seik Weng Ng

**Affiliations:** a Shanghai Sunvea Chemical Materials Science and Technology Co Ltd, Shanghai 201611, People’s Republic of China; b Institute of Marine Materials Science and Engineering, Shanghai Maritime University, Shanghai 201306, People’s Republic of China; c Department of Chemistry, University of Malaya, 50603 Kuala Lumpur, Malaysia

## Abstract

Bis[2-(4,6-dimethyl­pyrimidin-2-ylsulfan­yl)eth­yl]amine under hydro­thermal conditions has unexpectedly been transformed into the title compound, C_32_H_44_N_10_S_4_. In the title mol­ecule, the zigzag 3,10-diaza-6,7-disulfanyldodecyl skeleton has two dimethyl­pyrimidinylsulfanyl groups at both ends, and the aza atoms each carry a dimethyl­pyrimidinyl unit. The N atoms in the skeleton show a planar coordination.

## Related literature

For the crystal structures of ligands having two 4,6-dimethyl­pyridimin-2-ylsulfanyl units linked to a hydro­carbon chain, see: Chen *et al.* (2007 ▶); Wang *et al.* (2007 ▶); Wu *et al.* (2007*a* ▶,*b* ▶).
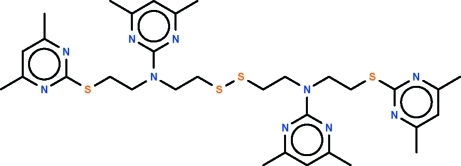


## Experimental

### 

#### Crystal data

C_32_H_44_N_10_S_4_
*M* *_r_* = 697.01
Triclinic, 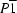

*a* = 11.7626 (5) Å
*b* = 12.7672 (6) Å
*c* = 13.7444 (7) Å
α = 106.382 (4)°
β = 103.276 (4)°
γ = 102.294 (4)°
*V* = 1840.15 (17) Å^3^
*Z* = 2
Cu *K*α radiation
μ = 2.67 mm^−1^
*T* = 293 K
0.30 × 0.25 × 0.20 mm

#### Data collection

Oxford Diffraction Xcalibur Sapphire 3 diffractometer
Absorption correction: multi-scan (*CrysAlis RED*; Oxford Diffraction, 2009 ▶) *T*_min_ = 0.947, *T*_max_ = 1.000
11434 measured reflections
7060 independent reflections
5177 reflections with *I* > 2σ(*I*)
*R*_int_ = 0.020

#### Refinement

*R*[*F* ^2^ > 2σ(*F* ^2^)] = 0.081
*wR*(*F* ^2^) = 0.252
*S* = 1.07
7060 reflections
423 parameters
4 restraints
H-atom parameters constrained
Δρ_max_ = 1.45 e Å^−3^
Δρ_min_ = −0.75 e Å^−3^

### 

Data collection: *CrysAlis PRO* (Oxford Diffraction, 2009 ▶); cell refinement: *CrysAlis PRO*; data reduction: *CrysAlis PRO*; program(s) used to solve structure: *SHELXS97* (Sheldrick, 2008 ▶); program(s) used to refine structure: *SHELXL97* (Sheldrick, 2008 ▶); molecular graphics: *X-SEED* (Barbour, 2001 ▶); software used to prepare material for publication: *publCIF* (Westrip, 2010 ▶).

## Supplementary Material

Crystal structure: contains datablocks global, I. DOI: 10.1107/S1600536810035245/xu5021sup1.cif

Structure factors: contains datablocks I. DOI: 10.1107/S1600536810035245/xu5021Isup2.hkl

Additional supplementary materials:  crystallographic information; 3D view; checkCIF report

## Figures and Tables

**Fig. 1.**
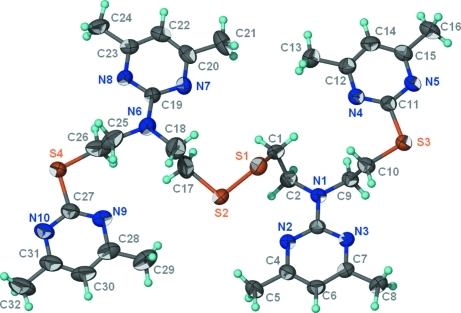
Thermal ellipsoid plot (Barbour, 2001) of C32H44N10S4 at the 50% probability level; hydrogen atoms are drawn as spheres of arbitrary radius.

## supplementary crystallographic information

### Comment

We are interested in synthesizing flexible ligands having two 4,6-dimethylpyridimin-2-ylsulfanyl units linked to a hydrocarbon chain; compounds such as 2,2'-bis(4,6-dimethylpyrimidn-2-ylsulfanyl)methane (Chen *et al.*, 2007) and its ethane (Wu *et al.*, 2007*b*), propane (Wu *et al.*, 2007*a*) and butane (Wang *et al.*, 2007) analogs have been synthesized for the purpose of studying its coordination chemistry in metal adducts. The coordination chemistry can be expanded in bis[2-(4,6-dimethylpyrimidin-2-ysulfanyl)ethyl]amine, whose synthesis has not been reported yet. However, the attempted complexation with copper ions under hydrothermal conditions yielded bis{2-[(4,6-dimethylpyrimidin-1-yl)(4,6-dimethylpyrimidin-1-ylsulfanyl-2-ethyl)amino]ethyl}disulfide (Scheme I, Fig. 1), a more interesting ligand whose exocyclic sulfur and endocyclic nitrogen sites offer many more sites for coordination.

### Experimental

Bis[2-(4,6-dimethylpyrimidin-2-ylsulfanyl)ethyl]amine was synthesized from the reaction of bis(2-chloroethyl)ammonium hydrochloride (1.78 g, 0.01 mol) dissolved in ethanol (100 ml) and 4,6-dimethylpyrimidine-2-thiol (2.80 g, 0.02 mol)/sodium hydroxide (0.8 g, 0.02 mol) dissolved in ethanol (200 ml). The solution was heated at 353 K for 8 h. The solvent was removed and the residue was column chromatographed with ethly acetate/petroleum ether (1/1 *v*/*v*) as eluent to yield a white powder; yield 63%. The formulation was confirmed by ^1^H NMR (CDCl_3_, 400 MHz) spectroscopy: 1.36–1.402(*m*, 1H), 2.274 (*d*,6*H*), 2.403–2.426 (*d*,6*H*), 2.813–2.871(*m*, 2H), 3.343–3.380 (*m*, 2H), 6.279 (*s*, 1H), 6.715 (*s*, 1H). This compound has not been reported in the chemical literature yet.

The title compound was the unexpected product obtained in the reaction of bis(2-(4,6-dimethylpyrimidin-2-ylthio)ethyl)amine (0.175 g, 0.5 mmol), copper perchlorate (0.132 g, 0.5 mmol) and water (8 ml). The reactants were heated in a 23-ml Teflon-lined Parr reactor at 413 K for 3 days. The mixture was cooled to room temperature at a rate of 5 K h^-1^. The prismatic crystals were collected and washed with water; yield: 40%. MS (ESI) m/*z*(%): 698.2 (*M*^+1^). CH&N elemental analysis, calculated for C_32_H_44_N_10_S_4_: C 55.14, H 6.36, N 20.09%. Found: C 55.50, H 6.56, N 19.56%.

### Refinement

Carbon-bound H-atoms were placed in calculated positions (C—H 0.93 to 0.96 Å) and were included in the refinement in the riding model approximation, with *U*(H) set to 1.2–1.5 times *U*_eq_(C). The final difference Fourier map had a peak in the vicinity of N6.

For the ethyl portions, the carbon-carbon distance was restrained to 1.53±0.01 Å.

### Crystal data

**Table:**

C_32_H_44_N_10_S_4_	*Z* = 2
*M**_r_* = 697.01	*F*(000) = 740
Triclinic, *P*1	*D*_x_ = 1.258 Mg m^−^^3^
Hall symbol: -P 1	Cu *K*α radiation, λ = 1.54184 Å
*a* = 11.7626 (5) Å	Cell parameters from 5344 reflections
*b* = 12.7672 (6) Å	θ = 4.6–74.5°
*c* = 13.7444 (7) Å	µ = 2.67 mm^−^^1^
α = 106.382 (4)°	*T* = 293 K
β = 103.276 (4)°	Prism, colorless
γ = 102.294 (4)°	0.30 × 0.25 × 0.20 mm
*V* = 1840.15 (17) Å^3^	

### Data collection

**Table:**

Oxford Diffraction Xcalibur Sapphire 3 diffractometer	7060 independent reflections
Radiation source: fine-focus sealed tube	5177 reflections with *I* > 2σ(*I*)
graphite	*R*_int_ = 0.020
Detector resolution: 16.0855 pixels mm^-1^	θ_max_ = 72.6°, θ_min_ = 4.6°
ω scans	*h* = −14→11
Absorption correction: multi-scan (*CrysAlis RED*; Oxford Diffraction, 2009)	*k* = −15→15
*T*_min_ = 0.947, *T*_max_ = 1.000	*l* = −16→16
11434 measured reflections	

### Refinement

**Table:**

Refinement on *F*^2^	Primary atom site location: structure-invariant direct methods
Least-squares matrix: full	Secondary atom site location: difference Fourier map
*R*[*F*^2^ > 2σ(*F*^2^)] = 0.081	Hydrogen site location: inferred from neighbouring sites
*wR*(*F*^2^) = 0.252	H-atom parameters constrained
*S* = 1.07	*w* = 1/[σ^2^(*F*_o_^2^) + (0.1399*P*)^2^ + 1.4306*P*] where *P* = (*F*_o_^2^ + 2*F*_c_^2^)/3
7060 reflections	(Δ/σ)_max_ = 0.001
423 parameters	Δρ_max_ = 1.45 e Å^−^^3^
4 restraints	Δρ_min_ = −0.75 e Å^−^^3^

### Fractional atomic coordinates and isotropic or equivalent isotropic displacement parameters (Å^2^)

**Table:**

	*x*	*y*	*z*	*U*_iso_*/*U*_eq_	
S1	0.74226 (10)	0.71528 (10)	0.39171 (9)	0.0696 (3)	
S2	0.57455 (12)	0.67895 (10)	0.41240 (10)	0.0812 (4)	
S3	0.96757 (10)	0.65326 (9)	0.03184 (10)	0.0661 (3)	
S4	0.36046 (14)	0.95980 (10)	0.78051 (12)	0.0903 (5)	
N1	0.6607 (3)	0.6061 (3)	0.1261 (3)	0.0538 (7)	
N2	0.5256 (3)	0.4838 (3)	0.1734 (3)	0.0527 (7)	
N3	0.6443 (3)	0.4147 (3)	0.0636 (2)	0.0531 (7)	
N4	0.9874 (3)	0.8593 (3)	0.1672 (3)	0.0582 (8)	
N5	1.0838 (3)	0.8487 (3)	0.0304 (3)	0.0598 (8)	
N6	0.6477 (4)	0.9727 (4)	0.6648 (4)	0.0905 (14)	
N7	0.7688 (3)	1.0764 (3)	0.5901 (3)	0.0745 (11)	
N8	0.6908 (4)	1.1703 (3)	0.7257 (3)	0.0685 (10)	
N9	0.3262 (4)	0.7490 (3)	0.6468 (3)	0.0712 (10)	
N10	0.1976 (4)	0.7779 (3)	0.7571 (3)	0.0731 (10)	
C1	0.7241 (4)	0.7789 (3)	0.2897 (3)	0.0645 (11)	
H1A	0.7028	0.8486	0.3162	0.077*	
H1B	0.8021	0.8000	0.2770	0.077*	
C2	0.6275 (4)	0.7027 (3)	0.1837 (3)	0.0590 (9)	
H2A	0.5514	0.6746	0.1972	0.071*	
H2B	0.6132	0.7485	0.1392	0.071*	
C3	0.6078 (3)	0.4974 (3)	0.1210 (3)	0.0484 (8)	
C4	0.4770 (3)	0.3770 (3)	0.1680 (3)	0.0554 (9)	
C5	0.3843 (5)	0.3613 (4)	0.2253 (4)	0.0772 (13)	
H5A	0.4117	0.4218	0.2932	0.116*	
H5B	0.3743	0.2891	0.2358	0.116*	
H5C	0.3075	0.3628	0.1837	0.116*	
C6	0.5088 (4)	0.2862 (3)	0.1110 (3)	0.0588 (9)	
H6	0.4739	0.2122	0.1075	0.071*	
C7	0.5939 (3)	0.3083 (3)	0.0593 (3)	0.0537 (9)	
C8	0.6332 (5)	0.2155 (4)	−0.0056 (4)	0.0706 (11)	
H8A	0.7200	0.2308	0.0227	0.106*	
H8B	0.6130	0.2129	−0.0782	0.106*	
H8C	0.5918	0.1434	−0.0031	0.106*	
C9	0.7418 (3)	0.6247 (3)	0.0630 (3)	0.0537 (9)	
H9A	0.7489	0.6996	0.0571	0.064*	
H9B	0.7049	0.5684	−0.0084	0.064*	
C10	0.8697 (3)	0.6174 (4)	0.1083 (3)	0.0591 (9)	
H10A	0.8643	0.5404	0.1081	0.071*	
H10B	0.9051	0.6693	0.1816	0.071*	
C11	1.0171 (3)	0.8040 (3)	0.0842 (3)	0.0551 (9)	
C12	1.0309 (4)	0.9735 (4)	0.2019 (4)	0.0638 (10)	
C13	0.9967 (5)	1.0376 (4)	0.2947 (5)	0.0839 (14)	
H13A	1.0156	1.0076	0.3511	0.126*	
H13B	0.9106	1.0291	0.2729	0.126*	
H13C	1.0418	1.1172	0.3196	0.126*	
C14	1.1014 (4)	1.0279 (4)	0.1525 (4)	0.0691 (11)	
H14	1.1308	1.1073	0.1768	0.083*	
C15	1.1274 (4)	0.9623 (4)	0.0662 (4)	0.0611 (10)	
C16	1.2025 (4)	1.0147 (4)	0.0081 (4)	0.0761 (13)	
H16A	1.1798	0.9639	−0.0643	0.114*	
H16B	1.2876	1.0277	0.0425	0.114*	
H16C	1.1883	1.0862	0.0088	0.114*	
C17	0.5658 (5)	0.8234 (6)	0.4963 (4)	0.112 (2)	
H17A	0.5790	0.8783	0.4604	0.134*	
H17B	0.4857	0.8149	0.5061	0.134*	
C18	0.6604 (5)	0.8636 (5)	0.5998 (4)	0.0878 (15)	
H18A	0.7409	0.8759	0.5906	0.105*	
H18B	0.6495	0.8076	0.6347	0.105*	
C19	0.7040 (5)	1.0778 (4)	0.6585 (4)	0.0789 (14)	
C20	0.8330 (4)	1.1790 (4)	0.5960 (3)	0.0659 (11)	
C21	0.9099 (5)	1.1770 (6)	0.5229 (5)	0.0984 (18)	
H21A	0.8579	1.1487	0.4503	0.148*	
H21B	0.9600	1.1280	0.5320	0.148*	
H21C	0.9613	1.2530	0.5393	0.148*	
C22	0.8282 (4)	1.2782 (4)	0.6648 (4)	0.0668 (11)	
H22	0.8742	1.3490	0.6685	0.080*	
C23	0.7540 (4)	1.2708 (3)	0.7285 (3)	0.0590 (9)	
C24	0.7415 (5)	1.3748 (4)	0.8051 (4)	0.0796 (14)	
H24A	0.6594	1.3786	0.7827	0.119*	
H24B	0.7977	1.4422	0.8062	0.119*	
H24C	0.7595	1.3701	0.8752	0.119*	
C25	0.5709 (5)	0.9658 (4)	0.7391 (4)	0.0811 (14)	
H25A	0.5534	0.8904	0.7449	0.097*	
H25B	0.6147	1.0219	0.8097	0.097*	
C26	0.4571 (4)	0.9884 (4)	0.6943 (5)	0.0888 (16)	
H26A	0.4172	0.9381	0.6208	0.107*	
H26B	0.4727	1.0671	0.6971	0.107*	
C27	0.2882 (4)	0.8113 (4)	0.7202 (4)	0.0642 (10)	
C28	0.2640 (5)	0.6370 (4)	0.6049 (4)	0.0784 (13)	
C29	0.3050 (7)	0.5657 (5)	0.5205 (5)	0.106 (2)	
H29A	0.3261	0.6081	0.4762	0.159*	
H29B	0.2399	0.4968	0.4776	0.159*	
H29C	0.3751	0.5468	0.5536	0.159*	
C30	0.1711 (6)	0.5938 (4)	0.6403 (5)	0.0937 (17)	
H30	0.1300	0.5159	0.6127	0.112*	
C31	0.1387 (5)	0.6670 (5)	0.7175 (5)	0.0860 (15)	
C32	0.0362 (7)	0.6270 (6)	0.7581 (7)	0.132 (3)	
H32A	0.0588	0.6671	0.8335	0.198*	
H32B	0.0197	0.5464	0.7445	0.198*	
H32C	−0.0357	0.6419	0.7225	0.198*	

### Atomic displacement parameters (Å^2^)

**Table:**

	*U*^11^	*U*^22^	*U*^33^	*U*^12^	*U*^13^	*U*^23^
S1	0.0649 (6)	0.0682 (7)	0.0666 (7)	0.0186 (5)	0.0204 (5)	0.0108 (5)
S2	0.0860 (8)	0.0554 (6)	0.0813 (8)	−0.0002 (5)	0.0436 (7)	−0.0057 (5)
S3	0.0617 (6)	0.0514 (5)	0.0835 (7)	0.0110 (4)	0.0360 (5)	0.0143 (5)
S4	0.0995 (9)	0.0567 (6)	0.1033 (10)	−0.0047 (6)	0.0636 (8)	0.0056 (6)
N1	0.0538 (17)	0.0440 (16)	0.0608 (18)	0.0087 (13)	0.0228 (15)	0.0141 (14)
N2	0.0541 (17)	0.0459 (16)	0.0562 (18)	0.0106 (13)	0.0209 (14)	0.0152 (13)
N3	0.0549 (17)	0.0450 (16)	0.0532 (17)	0.0090 (13)	0.0192 (14)	0.0104 (13)
N4	0.0518 (17)	0.0540 (18)	0.068 (2)	0.0145 (14)	0.0229 (15)	0.0174 (15)
N5	0.0507 (17)	0.0581 (19)	0.070 (2)	0.0102 (15)	0.0252 (16)	0.0213 (16)
N6	0.100 (3)	0.061 (2)	0.101 (3)	0.008 (2)	0.057 (3)	0.005 (2)
N7	0.066 (2)	0.075 (2)	0.067 (2)	0.0054 (18)	0.0332 (18)	0.0032 (18)
N8	0.079 (2)	0.0455 (17)	0.073 (2)	0.0055 (16)	0.0401 (19)	0.0054 (16)
N9	0.074 (2)	0.054 (2)	0.077 (2)	0.0105 (17)	0.0210 (19)	0.0160 (18)
N10	0.072 (2)	0.064 (2)	0.078 (2)	0.0025 (18)	0.0258 (19)	0.0287 (19)
C1	0.067 (2)	0.0390 (19)	0.082 (3)	0.0097 (17)	0.031 (2)	0.0108 (18)
C2	0.061 (2)	0.049 (2)	0.071 (2)	0.0184 (17)	0.0242 (19)	0.0219 (18)
C3	0.0443 (17)	0.0452 (18)	0.0489 (19)	0.0073 (14)	0.0111 (14)	0.0135 (15)
C4	0.051 (2)	0.055 (2)	0.056 (2)	0.0070 (16)	0.0172 (17)	0.0192 (17)
C5	0.080 (3)	0.067 (3)	0.096 (3)	0.015 (2)	0.048 (3)	0.033 (3)
C6	0.063 (2)	0.0431 (19)	0.066 (2)	0.0081 (17)	0.0184 (19)	0.0184 (17)
C7	0.054 (2)	0.0458 (19)	0.051 (2)	0.0107 (16)	0.0103 (16)	0.0107 (15)
C8	0.081 (3)	0.050 (2)	0.073 (3)	0.018 (2)	0.026 (2)	0.010 (2)
C9	0.054 (2)	0.051 (2)	0.057 (2)	0.0110 (16)	0.0187 (17)	0.0218 (17)
C10	0.056 (2)	0.055 (2)	0.068 (2)	0.0122 (17)	0.0220 (19)	0.0270 (19)
C11	0.0441 (18)	0.054 (2)	0.065 (2)	0.0122 (16)	0.0185 (17)	0.0168 (18)
C12	0.054 (2)	0.056 (2)	0.072 (3)	0.0145 (18)	0.0188 (19)	0.012 (2)
C13	0.083 (3)	0.068 (3)	0.095 (4)	0.020 (2)	0.041 (3)	0.011 (3)
C14	0.061 (2)	0.047 (2)	0.087 (3)	0.0054 (18)	0.020 (2)	0.016 (2)
C15	0.048 (2)	0.062 (2)	0.073 (3)	0.0114 (17)	0.0171 (18)	0.027 (2)
C16	0.064 (3)	0.078 (3)	0.089 (3)	0.007 (2)	0.028 (2)	0.040 (3)
C17	0.074 (3)	0.142 (6)	0.083 (4)	0.004 (3)	0.042 (3)	−0.008 (4)
C18	0.099 (4)	0.084 (3)	0.088 (4)	0.034 (3)	0.035 (3)	0.031 (3)
C19	0.082 (3)	0.056 (2)	0.085 (3)	0.000 (2)	0.047 (3)	0.002 (2)
C20	0.046 (2)	0.090 (3)	0.061 (2)	0.012 (2)	0.0162 (18)	0.032 (2)
C21	0.078 (3)	0.140 (5)	0.105 (4)	0.038 (3)	0.053 (3)	0.059 (4)
C22	0.054 (2)	0.064 (3)	0.078 (3)	0.0054 (19)	0.015 (2)	0.031 (2)
C23	0.055 (2)	0.050 (2)	0.061 (2)	0.0077 (17)	0.0109 (18)	0.0150 (17)
C24	0.075 (3)	0.044 (2)	0.100 (4)	0.008 (2)	0.020 (3)	0.009 (2)
C25	0.122 (4)	0.055 (2)	0.060 (3)	0.028 (3)	0.016 (3)	0.018 (2)
C26	0.071 (3)	0.058 (3)	0.124 (5)	0.009 (2)	0.011 (3)	0.036 (3)
C27	0.064 (2)	0.054 (2)	0.069 (3)	0.0066 (19)	0.021 (2)	0.022 (2)
C28	0.082 (3)	0.053 (2)	0.088 (3)	0.014 (2)	0.013 (3)	0.022 (2)
C29	0.125 (5)	0.063 (3)	0.107 (4)	0.026 (3)	0.023 (4)	0.008 (3)
C30	0.104 (4)	0.050 (3)	0.104 (4)	−0.004 (3)	0.019 (3)	0.024 (3)
C31	0.088 (3)	0.065 (3)	0.091 (4)	−0.005 (3)	0.022 (3)	0.032 (3)
C32	0.127 (6)	0.103 (5)	0.149 (6)	−0.024 (4)	0.059 (5)	0.048 (5)

### Geometric parameters (Å, °)

**Table:**

S1—C1	1.802 (5)	C9—H9B	0.9700
S1—S2	2.0323 (17)	C10—H10A	0.9700
S2—C17	1.918 (7)	C10—H10B	0.9700
S3—C11	1.764 (4)	C12—C14	1.382 (6)
S3—C10	1.801 (4)	C12—C13	1.499 (6)
S4—C27	1.763 (4)	C13—H13A	0.9600
S4—C26	1.874 (6)	C13—H13B	0.9600
N1—C3	1.369 (5)	C13—H13C	0.9600
N1—C2	1.449 (5)	C14—C15	1.385 (6)
N1—C9	1.456 (5)	C14—H14	0.9300
N2—C4	1.335 (5)	C15—C16	1.497 (6)
N2—C3	1.343 (5)	C16—H16A	0.9600
N3—C7	1.339 (5)	C16—H16B	0.9600
N3—C3	1.342 (5)	C16—H16C	0.9600
N4—C11	1.324 (5)	C17—C18	1.464 (6)
N4—C12	1.339 (5)	C17—H17A	0.9700
N5—C15	1.331 (5)	C17—H17B	0.9700
N5—C11	1.343 (5)	C18—H18A	0.9700
N6—C19	1.399 (6)	C18—H18B	0.9700
N6—C18	1.486 (7)	C20—C22	1.371 (6)
N6—C25	1.520 (7)	C20—C21	1.497 (6)
N7—C20	1.336 (6)	C21—H21A	0.9600
N7—C19	1.338 (6)	C21—H21B	0.9600
N8—C23	1.325 (5)	C21—H21C	0.9600
N8—C19	1.342 (5)	C22—C23	1.378 (6)
N9—C27	1.324 (6)	C22—H22	0.9300
N9—C28	1.345 (6)	C23—C24	1.503 (6)
N10—C27	1.324 (6)	C24—H24A	0.9600
N10—C31	1.325 (6)	C24—H24B	0.9600
C1—C2	1.526 (5)	C24—H24C	0.9600
C1—H1A	0.9700	C25—C26	1.464 (6)
C1—H1B	0.9700	C25—H25A	0.9700
C2—H2A	0.9700	C25—H25B	0.9700
C2—H2B	0.9700	C26—H26A	0.9700
C4—C6	1.380 (6)	C26—H26B	0.9700
C4—C5	1.498 (6)	C28—C30	1.369 (8)
C5—H5A	0.9600	C28—C29	1.501 (8)
C5—H5B	0.9600	C29—H29A	0.9600
C5—H5C	0.9600	C29—H29B	0.9600
C6—C7	1.380 (6)	C29—H29C	0.9600
C6—H6	0.9300	C30—C31	1.387 (8)
C7—C8	1.497 (5)	C30—H30	0.9300
C8—H8A	0.9600	C31—C32	1.495 (8)
C8—H8B	0.9600	C32—H32A	0.9600
C8—H8C	0.9600	C32—H32B	0.9600
C9—C10	1.524 (5)	C32—H32C	0.9600
C9—H9A	0.9700		
			
C1—S1—S2	104.28 (16)	N5—C15—C14	120.6 (4)
C17—S2—S1	104.44 (18)	N5—C15—C16	117.3 (4)
C11—S3—C10	102.6 (2)	C14—C15—C16	122.1 (4)
C27—S4—C26	102.5 (2)	C15—C16—H16A	109.5
C3—N1—C2	121.5 (3)	C15—C16—H16B	109.5
C3—N1—C9	119.8 (3)	H16A—C16—H16B	109.5
C2—N1—C9	118.3 (3)	C15—C16—H16C	109.5
C4—N2—C3	116.0 (3)	H16A—C16—H16C	109.5
C7—N3—C3	116.3 (3)	H16B—C16—H16C	109.5
C11—N4—C12	115.6 (4)	C18—C17—S2	108.2 (5)
C15—N5—C11	116.0 (4)	C18—C17—H17A	110.0
C19—N6—C18	121.8 (4)	S2—C17—H17A	110.0
C19—N6—C25	121.0 (4)	C18—C17—H17B	110.0
C18—N6—C25	117.2 (4)	S2—C17—H17B	110.0
C20—N7—C19	115.3 (4)	H17A—C17—H17B	108.4
C23—N8—C19	116.3 (4)	C17—C18—N6	107.1 (5)
C27—N9—C28	115.1 (4)	C17—C18—H18A	110.3
C27—N10—C31	115.9 (5)	N6—C18—H18A	110.3
C2—C1—S1	114.9 (3)	C17—C18—H18B	110.3
C2—C1—H1A	108.5	N6—C18—H18B	110.3
S1—C1—H1A	108.5	H18A—C18—H18B	108.6
C2—C1—H1B	108.5	N7—C19—N8	126.8 (4)
S1—C1—H1B	108.5	N7—C19—N6	117.4 (4)
H1A—C1—H1B	107.5	N8—C19—N6	115.7 (4)
N1—C2—C1	113.8 (3)	N7—C20—C22	121.7 (4)
N1—C2—H2A	108.8	N7—C20—C21	115.1 (5)
C1—C2—H2A	108.8	C22—C20—C21	123.2 (5)
N1—C2—H2B	108.8	C20—C21—H21A	109.5
C1—C2—H2B	108.8	C20—C21—H21B	109.5
H2A—C2—H2B	107.7	H21A—C21—H21B	109.5
N3—C3—N2	126.5 (3)	C20—C21—H21C	109.5
N3—C3—N1	116.0 (3)	H21A—C21—H21C	109.5
N2—C3—N1	117.5 (3)	H21B—C21—H21C	109.5
N2—C4—C6	121.8 (4)	C20—C22—C23	118.6 (4)
N2—C4—C5	116.2 (4)	C20—C22—H22	120.7
C6—C4—C5	122.0 (4)	C23—C22—H22	120.7
C4—C5—H5A	109.5	N8—C23—C22	121.0 (4)
C4—C5—H5B	109.5	N8—C23—C24	116.6 (4)
H5A—C5—H5B	109.5	C22—C23—C24	122.4 (4)
C4—C5—H5C	109.5	C23—C24—H24A	109.5
H5A—C5—H5C	109.5	C23—C24—H24B	109.5
H5B—C5—H5C	109.5	H24A—C24—H24B	109.5
C4—C6—C7	118.2 (4)	C23—C24—H24C	109.5
C4—C6—H6	120.9	H24A—C24—H24C	109.5
C7—C6—H6	120.9	H24B—C24—H24C	109.5
N3—C7—C6	121.3 (3)	C26—C25—N6	107.6 (4)
N3—C7—C8	116.5 (4)	C26—C25—H25A	110.2
C6—C7—C8	122.2 (4)	N6—C25—H25A	110.2
C7—C8—H8A	109.5	C26—C25—H25B	110.2
C7—C8—H8B	109.5	N6—C25—H25B	110.2
H8A—C8—H8B	109.5	H25A—C25—H25B	108.5
C7—C8—H8C	109.5	C25—C26—S4	104.7 (4)
H8A—C8—H8C	109.5	C25—C26—H26A	110.8
H8B—C8—H8C	109.5	S4—C26—H26A	110.8
N1—C9—C10	114.3 (3)	C25—C26—H26B	110.8
N1—C9—H9A	108.7	S4—C26—H26B	110.8
C10—C9—H9A	108.7	H26A—C26—H26B	108.9
N1—C9—H9B	108.7	N10—C27—N9	128.7 (4)
C10—C9—H9B	108.7	N10—C27—S4	111.4 (3)
H9A—C9—H9B	107.6	N9—C27—S4	119.9 (3)
C9—C10—S3	111.4 (3)	N9—C28—C30	120.5 (5)
C9—C10—H10A	109.4	N9—C28—C29	115.7 (5)
S3—C10—H10A	109.4	C30—C28—C29	123.8 (5)
C9—C10—H10B	109.4	C28—C29—H29A	109.5
S3—C10—H10B	109.4	C28—C29—H29B	109.5
H10A—C10—H10B	108.0	H29A—C29—H29B	109.5
N4—C11—N5	127.8 (4)	C28—C29—H29C	109.5
N4—C11—S3	120.2 (3)	H29A—C29—H29C	109.5
N5—C11—S3	111.9 (3)	H29B—C29—H29C	109.5
N4—C12—C14	121.1 (4)	C28—C30—C31	119.5 (5)
N4—C12—C13	116.3 (4)	C28—C30—H30	120.2
C14—C12—C13	122.6 (4)	C31—C30—H30	120.2
C12—C13—H13A	109.5	N10—C31—C30	120.3 (5)
C12—C13—H13B	109.5	N10—C31—C32	117.0 (6)
H13A—C13—H13B	109.5	C30—C31—C32	122.8 (5)
C12—C13—H13C	109.5	C31—C32—H32A	109.5
H13A—C13—H13C	109.5	C31—C32—H32B	109.5
H13B—C13—H13C	109.5	H32A—C32—H32B	109.5
C12—C14—C15	118.9 (4)	C31—C32—H32C	109.5
C12—C14—H14	120.6	H32A—C32—H32C	109.5
C15—C14—H14	120.6	H32B—C32—H32C	109.5
			
C1—S1—S2—C17	−77.4 (3)	S1—S2—C17—C18	−63.6 (5)
S2—S1—C1—C2	−59.5 (3)	S2—C17—C18—N6	−177.4 (4)
C3—N1—C2—C1	105.5 (4)	C19—N6—C18—C17	−85.7 (7)
C9—N1—C2—C1	−81.3 (4)	C25—N6—C18—C17	94.5 (6)
S1—C1—C2—N1	−69.4 (4)	C20—N7—C19—N8	5.0 (8)
C7—N3—C3—N2	−0.5 (6)	C20—N7—C19—N6	−172.1 (5)
C7—N3—C3—N1	179.3 (3)	C23—N8—C19—N7	−4.0 (8)
C4—N2—C3—N3	0.7 (6)	C23—N8—C19—N6	173.2 (5)
C4—N2—C3—N1	−179.1 (3)	C18—N6—C19—N7	1.0 (8)
C2—N1—C3—N3	178.8 (3)	C25—N6—C19—N7	−179.2 (5)
C9—N1—C3—N3	5.8 (5)	C18—N6—C19—N8	−176.5 (5)
C2—N1—C3—N2	−1.3 (5)	C25—N6—C19—N8	3.4 (8)
C9—N1—C3—N2	−174.3 (3)	C19—N7—C20—C22	−2.4 (7)
C3—N2—C4—C6	−0.6 (6)	C19—N7—C20—C21	177.2 (5)
C3—N2—C4—C5	−179.4 (4)	N7—C20—C22—C23	−0.7 (7)
N2—C4—C6—C7	0.4 (6)	C21—C20—C22—C23	179.7 (4)
C5—C4—C6—C7	179.1 (4)	C19—N8—C23—C22	0.4 (7)
C3—N3—C7—C6	0.3 (5)	C19—N8—C23—C24	−178.9 (4)
C3—N3—C7—C8	179.3 (3)	C20—C22—C23—N8	1.8 (7)
C4—C6—C7—N3	−0.2 (6)	C20—C22—C23—C24	−179.1 (4)
C4—C6—C7—C8	−179.2 (4)	C19—N6—C25—C26	71.8 (6)
C3—N1—C9—C10	−77.2 (4)	C18—N6—C25—C26	−108.3 (5)
C2—N1—C9—C10	109.6 (4)	N6—C25—C26—S4	173.0 (3)
N1—C9—C10—S3	−175.1 (3)	C27—S4—C26—C25	−86.1 (4)
C11—S3—C10—C9	82.3 (3)	C31—N10—C27—N9	1.8 (8)
C12—N4—C11—N5	−0.9 (6)	C31—N10—C27—S4	−179.1 (4)
C12—N4—C11—S3	179.1 (3)	C28—N9—C27—N10	−0.2 (7)
C15—N5—C11—N4	1.5 (6)	C28—N9—C27—S4	−179.3 (4)
C15—N5—C11—S3	−178.5 (3)	C26—S4—C27—N10	−167.7 (3)
C10—S3—C11—N4	5.5 (4)	C26—S4—C27—N9	11.5 (4)
C10—S3—C11—N5	−174.5 (3)	C27—N9—C28—C30	−1.7 (7)
C11—N4—C12—C14	0.4 (6)	C27—N9—C28—C29	179.1 (5)
C11—N4—C12—C13	179.1 (4)	N9—C28—C30—C31	2.0 (9)
N4—C12—C14—C15	−0.4 (7)	C29—C28—C30—C31	−178.9 (5)
C13—C12—C14—C15	−179.1 (4)	C27—N10—C31—C30	−1.4 (8)
C11—N5—C15—C14	−1.4 (6)	C27—N10—C31—C32	179.8 (6)
C11—N5—C15—C16	179.8 (4)	C28—C30—C31—N10	−0.3 (9)
C12—C14—C15—N5	1.0 (7)	C28—C30—C31—C32	178.4 (6)
C12—C14—C15—C16	179.7 (4)		
